# Woven Natural Fibre Reinforced Composite Materials for Medical Imaging

**DOI:** 10.3390/ma13071684

**Published:** 2020-04-04

**Authors:** Robert H. Morris, Nicasio R. Geraldi, Johanna L. Stafford, Abi Spicer, James Hall, Christopher Bradley, Michael I. Newton

**Affiliations:** 1 School of Science and Technology, Nottingham Trent University, Nottingham NG11 8NS, UK; nicasio.geraldi02@ntu.ac.uk (N.R.G.); johanna.stafford2016@my.ntu.ac.uk (J.L.S.); abi.spicer02@ntu.ac.uk (A.S.); jim.hall@ntu.ac.uk (J.H.); michael.newton@ntu.ac.uk (M.I.N.); 2 Faculty of Science, University of Nottingham, Nottingham NG7 2RD, UK; christopher.bradley@nottingham.ac.uk

**Keywords:** natural fibre composite, magnetic resonance imaging, radiotherapy, X-Ray, woven, medical imaging

## Abstract

Repeatable patient positioning is key to minimising the burden on planning radiotherapy treatment. There are very few materials commercially available which are suitable for use in all common imaging and treatment modalities such as magnetic resonance imaging (MRI), X-Ray computed tomography (CT) and radiotherapy. In this article, we present several such materials based on woven natural fibres embedded in a range of different resin materials which are suitable for such applications. By investigating a range of resins and natural fibre materials in combination and evaluating their performance in terms of MRI and X-Ray imaging, we show that a woven cotton material impregnated with a two-part epoxy resin provides a 15% improvement in passage of X-Rays and has no impact on the MRI signal (unlike the 40% MRI signal attenuation from carbon fibre), whilst also retaining a flexural modulus up to 71% of that of carbon fibre. These results demonstrate that natural fibre composites produced using such materials provide desirable properties for use in patient support and positioning devices for multi-modal imaging, without the need to significantly compromise on the strength of the material.

## 1. Introduction

As cancer treatments are becoming increasingly advanced, there is a growing demand for treatment plans to be based upon images from more than one imaging modality [1,2]. The repeatable positioning of patients in the different modalities has required that flat patient supports, also referred to in the literature as table tops, be made available for the range of different manufacturers’ machines. These supports are optimised for the individual modalities, meaning that the same patient support cannot simply be moved between machines which would allow for optimum positioning. In addition, they must have sufficient strength and be light enough for safe and convenient operator removal. Current materials used in the production of patient supports include carbon fibre and glass fibre composites [3,4]. In a recent article [5] we presented a composite comprising wood pulp-derived fibres embedded in polyester resin and bonded to expanded polystyrene cores. We compared these to the standards of glass fibre reinforced honeycomb cored composite (GFC) and carbon fibre reinforced composite (CFC). The new material outperformed them both in terms of Magnetic Resonance Imaging (MRI) Radio Frequency (RF) shading and distortion. It gave similar performance to the CFC with regard to megavoltage imaging homogeneity and outperformed the GFC by 56%. This natural fibre-based composite had somewhat poorer performance in mechanical testing but still performed within the required limits. It should be noted that contact between the patient and these materials is minimal since they are typically covered in a disposable paper roll which is changed between the patients who are typically clothed. Where contact is expected, it is typically less than 30 minutes.

Composites are an important class of materials which by definition are formed by the combination of two dissimilar materials to produce a new, with better properties than the individual components. The most common composite materials are formed by combining a thermoset resin, such as a two-part epoxy, as a binder, with some form of filler as a matrix. There are, however, a wide range of composite materials known and commercially available, ranging from cement binders loaded with amorphous SiO_2_ nanospheres [6] or fly ash [7] for use in construction, down to composite bearings for machine tooling (miler). Composites are also used to replace components, such as the bearings in the last example, in environments where corrosion of metals would normally limit use, such as in sea water [8]. Composites can also be produced from waste materials such as earthquake resistance isolators produced using fibre reinforced recycled rubber [9]. Within a clinical setting, composites have been replacing their ceramic counterparts for a number of decades in dental work [10] and in more recent years for the production of hip replacement hip implants [11]. 

With an ever-growing focus on the environmental impact of materials and processes, using natural fibres as the filler or matrix in composite materials has become more commonplace. Natural fibres can be plant based, such as flax, jute, cotton, or bamboo, or animal derived, such as silk and wool. Many natural fibres have drawbacks, such as being less homogeneous than glass or carbon fibres, higher in moisture absorption and lower compatibility with conventional resins [12,13,14,15,16]. Most natural fibres are, however, more eco-friendly and biodegradable than their synthetic counterparts.

Unlike other studies into natural fibre composites, we focus on the need to reduce x-ray attenuation and maintain MRI compatibility so we expect to require some compromise on mechanical metrics. In this article we investigated a range of natural fibres combined with an ultraviolet (UV) photocuring soy-based resin, which we compare to conventional epoxy and a fully synthetic (UV) photocuring resin to determine the viability of creating a composite material based on natural components.

## 2. Materials and Methods

A full assessment of the trade-off between imaging performance and mechanical properties was undertaken on a range of samples. The steps needed to process the materials and the techniques used to assess their performance are detailed in this section.

### 2.1. Material Processing

A range of sheet materials produced from natural fibres were acquired to allow for the influence of the composition to be determined. Materials tested are detailed in Table 1. A brushed cotton was initially also tested but the composites which it produced were far too thick to be of relevance for such a product. A range of coatings were applied to such materials during production to achieve desirable qualities for the end user which interfered with the process of creating composite materials. To address this, all materials were boiled in tap water for 10 minutes. These sheet materials all had relatively tight weaves with varying levels of fibre smoothness. The ultimate strength of the composite material was dependent on a number of factors, including: the quantity of resin per unit area; the extent to which the resin keyed into the fibres (which increased with fibre roughness); and the overall strength of the fibre reinforcement. To improve the ability of the resin to penetrate into the fibre matrix, we investigated the use of a sodium hydroxide solution to modify the surface structure of the fabric sheets (known in the textile industry as mercerization) based on two methods in the literature [17,18]. A highly concentrated solution (4.5 mol.dm^−3^) was made up with water in which the range of materials was bathed for either 30 seconds or between 1 and 5 minutes in 1-minute intervals. The effect on the fibres was then determined using a tensile test and scanning electron microscopy. For comparison purposes, a sample of carbon fibre composite material was also prepared using the epoxy resin (since UV curing is not possible with black opaque materials).

### 2.2. Assessment of Fabric Properties

Scanning electron microscopy was undertaken (Model Number JSM-7100F, Jeol, Tokyo, Japan) for 10mmx10mm squares of each material which were mounted onto 10mm round stubs with carbon tape and sputter coated (Q150R ES, Quorum Technologies Ltd, Sussex, UK) with 4nm of gold. Each stub was in turn placed into the Scanning Electron Microscope (SEM) chamber and imaged at 100× magnification. Images were acquired with an isotropic resolution of 1μm.

The tensile strength of 30mm wide samples, prepared in accordance with ISO standard 13924, was tested for elongation and breaking force using an automated testing rig (6000R, Lloyd Instruments, Hampshire, UK) with a 3 kN head (NLC, Lloyd Instruments, Hampshire, UK). Data were acquired using a USB multifunction I/O computer interface (USB-6210, National Instruments, Austin, TX, USA) with a custom software interface to log the results (produced in LabView, National Instruments, Austin, TX, USA). Samples were acquired with 14-bit precision with full scale represented by the maximum load of the head. Thus, for these measurements, we had a resolution of 18 mN. A single sodium hydroxide processing time was then selected for each material based upon the balance between increasing fibre roughness and reducing tensile strength. Samples were tested in triplicate.

### 2.3. Composite Creation

Composite sheets were then created from each of the natural fibre composites which had been boiled for 10 minutes in tap water and also for sheets processed in sodium hydroxide for a time as determined in the previous section. Four layers of the natural fibre sheet of interest were placed on a flat high-pressure laminate surface which had been waxed using Meguiar’s #8 Mirror Glaze Mould Release Wax (EasyComposites, Staffordshire, UK) for epoxy resins or glass for UV curing resins. A layer each of peel ply, infusion mesh and single use vacuum bag film (PP230 Aero-Grade Nylon 66, FM100 Infusion Mesh and VB200 Vacuum Bagging Film respectively, all Easy Composites, Staffordshire, UK) were placed on top and sealed to the high pressure laminate using gum tape. For the epoxy samples, epoxy resin was then mixed with a fast hardener as detailed in Table 2 and drawn into the dry layup using a vacuum pump (6i, Edwards, West Sussex, UK). Once the resin had permeated all of the fabric, the inlet and outlet were clamped off and the system was left to cure for 24 hours at room temperature. For the UV samples, either white synthetic based resin or a soy-based resin, also detailed in Table 2, was drawn into the fabric in the same way without the use of infusion mesh to allow for maximum penetration of UV light. After clamping, it was cured for 10 minutes under ultraviolet light using a custom-built assembly of 6 UV tubes (TLAD 15W/05, Phillips, Netherlands) while held under vacuum. These processes resulted in a composite material in which the natural fibres were fully sealed. Final products would however ultimately be sealed with a non-porous lacquer for the purposes of infection control. 

### 2.4. Evaluation of Mechanical Properties

The mechanical properties of the resulting composite skins were tested using the same testing machine as for fabric evaluation in compression mode with a 20N head (DLC, Lloyd Instruments, Hampshire, UK) using a standardised 3-point bend fixture. The Flexural modulus is calculated according to Equation (1).

(1) $$E_{f}=\frac{L^{3}m}{4bd^{3}}$$

where *L, m, b* and *d* are the support span (50 mm with 10 mm diameter soft points), the gradient of the linear portion of the deflection curve, the width of the test sheet (20 mm) and the thickness of the test sheet, respectively. The higher the flexural modulus, the stiffer the material. Samples were tested in duplicate from different areas of the sample.

### 2.5. Clinical Imaging Assessment

Images of 6-well plates (140675, Sarstedt, Nümbrecht) with each well placed above a different sample of composite skin (or without composite in the case of controls) were acquired using a Philips Achieva 3T MRI system and a Siemens Avanto 1.5T MRI system. The wells were filled with 2ml of fluid as used in the ACR phantom [19] (a common calibration phantom used for MRI which replicated the properties of human tissue in terms of conductivity and relativity) and imaged coronally. The signal from each well was divided by the signal in the absence of sample to provide a ratio. If there was any distortion or signal loss caused by the material, then this would be less than 1 (within error). Samples were tested in duplicate.

X-ray attenuation was assessed using a benchtop X-ray imaging system (554 801 with 554 821, Leybold UK ltd, Chessington, UK) at 35 kV and 1mA filament current. Samples of 2 cm width were stacked in a stepped arrangement with heights from 3 to 6 cm to provide a range of thicknesses in a single image. The mean signal from each of the overlapping materials was used to determine the attenuation coefficient as the gradient of the signal intensity against the natural log of the thickness. Error bars represented the mean plus and minus 5 standard deviations within the measurement area. Data were collected and averaged from pixel values in a wide strip encompassing 80% of the sample width for samples in duplicate.

## 3. Results

### 3.1. SEM

**Figure 1:**
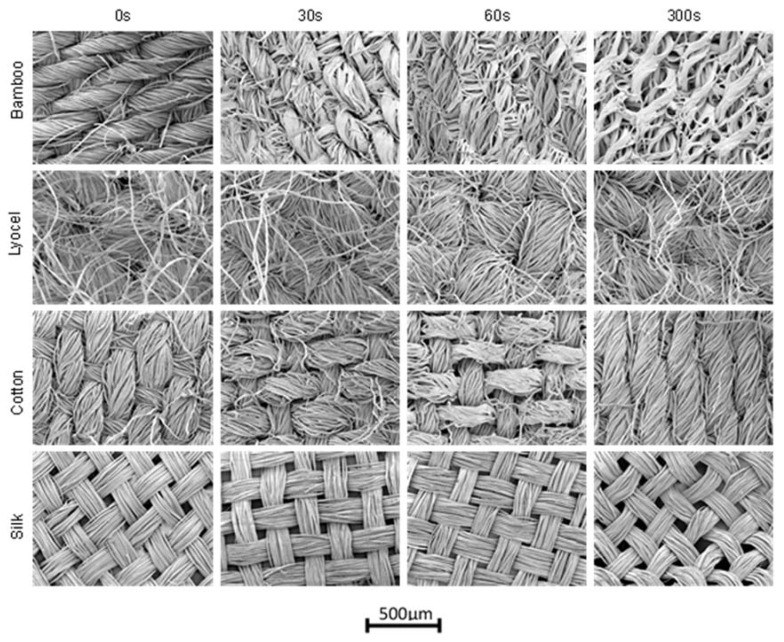
Time course of the effect of sodium hydroxide on natural fibres. Labels on the left refer to all images in that row. The time in seconds of exposure detailed across the top of the figure refer to all images in that column. The red scale bar in the bottom right represents a length of 500μm on the images, all of which have the same scale.

The SEM images for each of the fibre types are collected (SEM Model Number JSM-7100F, Jeol, Tokyo, Japan) and shown in Figure 1 for immersion times of 30 s, 2 minutes and 5 minutes in sodium hydroxide to demonstrate the evolution of the matrix over time. All images are shown at 100× magnification to provide an overview of both the bulk surface topography and the morphology of the individual yarns that comprise the fabrics.

It can be seen from Figure 1 that there is a variable effect of sodium hydroxide, with the most pronounced effect present on the bamboo fibres while silk experiences negligible change. Where changes are seen, they are similar between fibres within an individual yarn; initially swelling then appearing to merge together, ultimately eroding. The effect on the tensile strength of this process was then investigated.

### 3.2. Fabric Tensile Testing

**Figure 2:**
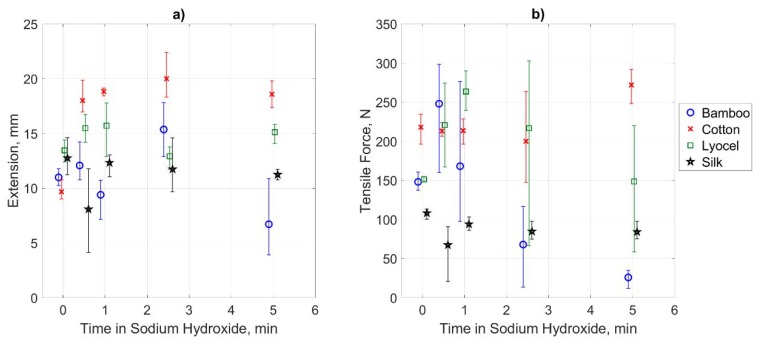
(**a**) Breaking force of fabric samples as a function of time in sodium hydroxide. (**b**) Elongation at breaking force.

The results of the tensile testing are shown in Figure 2, with range bars for the triplicate samples. This verifies the findings of the SEM imaging with a variable effect on the fibre strength. The bamboo, as expected from the significant change in morphology, sees the greatest change in tensile strength which initially increases before significantly falling to become the weakest sample. Its ductility does not change significantly as indicated by relatively constant extension at break. The cotton has a consistent and significant breaking strength, whilst the silk has a consistent but poor breaking strength. The ductility of the cotton is however, almost doubled by treatment with sodium hydroxide for any duration, whilst the silk retains a similar ductility. Finally, the lyocel sample experiences an increased breaking force for intermediate treatment times and retains similar ductility throughout.

Based on these findings, the bamboo, cotton, lyocel and silk will be treated for 30 seconds, 300 seconds, 60 seconds and 30 seconds, respectively, in the following sample production. 

### 3.3. Three Point Bend Test of Skins

**Figure 3:**
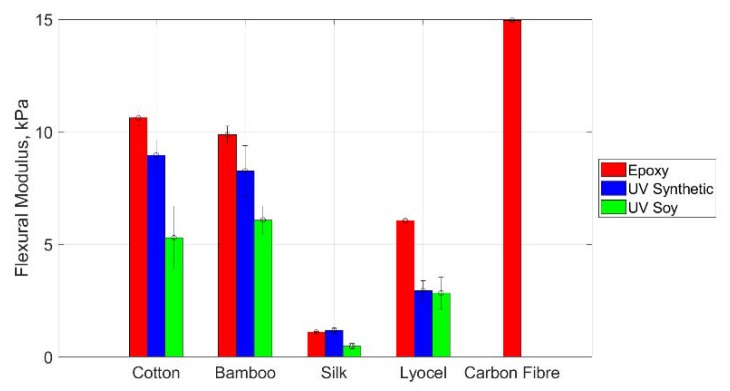
Comparison of the flexural modulus of samples produced using different resin and matrix types. Range bars are presented based on measurements from all samples in that class, e.g., the average and range bars on the epoxy cotton data are based on measurements of both repeats of both weave directions with and without sodium hydroxide treatment, representing eight samples.

The full three-point bend test results are shown in supplementary information Figure S1. There was no significant difference between the samples which were treated in sodium hydroxide and those which were not. This would suggest that the role of the fibre morphology has little impact on the resulting flexural modulus. As such, the two data sets are averaged to provide greater ease in interpreting other findings, the results of which are shown in Figure 3. It can be seen that there is a dependence of the flexural modulus primarily on resin type with epoxy resin yielding the highest flexural modulus followed by the synthetic UV resin, followed by the soy-based UV resin in all cases other than for the silk. In this case, however, the resulting materials are all very thin in comparison to the other fabric types, resulting in less overall difference between the samples. The relationship between these values is not significantly influenced by dividing them by the sample density, suggesting that the overall stiffness is indeed due to the interplay between the fabric matrix and resin and not simply dependent on the quantity of the resin. The carbon fibre has a higher flexural modulus than the other materials, with the next highest coming from the epoxy cotton material at 71% of its value. Range bars are shown in the figure, encompassing all readings for that class of sample to highlight the variation which is experienced.

### 3.4. Clinical Imaging

**Figure 4:**
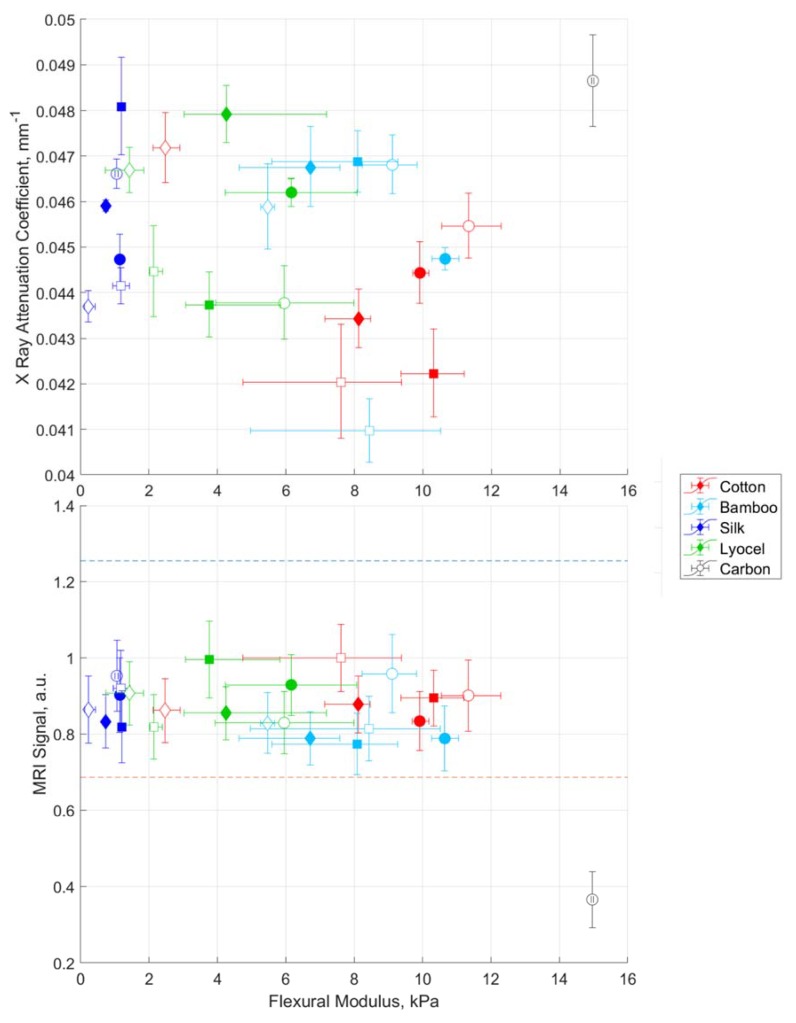
Overview of performance for X-ray (top) and magnetic resonance imaging (MRI) (bottom) as a function of flexural modulus. The optimum position for X-Ray is bottom right, the optimum position for MRI is top right. Colours represent different fibres as detailed in the legend. The shapes represent different resins as follows: circles (O) epoxy, squares (□) synthetic UV, diamond (◇) soy UV. Finally, untreated are open and treated are filled.

The results of the clinical imaging assessments are plotted against flexural modulus in Figure 4 to allow their overall performance to be predicted for clinical use. The most desirable position for the CT data plotted in Figure 4a is the bottom right hand corner, which represents a material with high flexural modulus and minimal X-Ray attenuation. It can be seen that all samples, with the exception of soy resin with a silk matrix, outperform carbon fibre in terms of X-Ray translucency. The epoxy cotton composites offer the maximum strength, while the minimal attenuation is seen for the synthetic UV resin with bamboo. 

The results of the MRI experiments at 1.5T are plotted in Figure 4b again against the flexural modulus. The optimum position on this plot is the top right-hand corner, representing minimal attenuation of the MRI signal as a result of RF shading with the highest flexural modulus. The dotted lines on the plot represent the range of values which have been found for the control samples owing to the spatial variation in the RF and static magnetic field homogeneities. It can be seen that all natural fibre composite samples fall within this range, demonstrating the minimal effect which it has on the RF reaching the fluid. The carbon fibre, on the other hand, is below the minimum value, demonstrating that it is less suitable for MRI imaging. The results from the 3T imaging system are shown in supplementary information Figure S2. Although they have more scatter than the 1.5T system, owing to greater distortion of the static magnetic field over the imaging region, the natural fibre composites all fall within the range of the controls in contrast to the carbon fibre. 

## 4. Discussion

There is some correlation between the breaking force of the unimpregnated fabrics and the resulting flexural moduli of the composites. The variance is, however, too high to be able to accurately predict the behaviour of the final composite. These data are shown in the supplementary material Figure S3. In terms of medical imaging performance, all-natural fibre composite materials which have been produced, with the exception of the soy resin in silk, outperform the carbon fibre samples in terms of X-Ray attenuation and MRI RF shading. The flexural modulus of the carbon fibre exceeds that of all-natural fibre composites. The epoxy cotton provides the highest flexural modulus of the natural fibre composites at 71% of the value of carbon fibre. There is little to distinguish the performance of the bamboo and cotton composites, thus for practical applications the trade-off between strength and X-ray performance must be individually determined.

The optimum materials for use are either epoxy-impregnated cotton, offering the highest flexural modulus, or synthetic UV resin-impregnated bamboo, which offers the minimum X-Ray attenuation without significant compromise of flexural modulus. Further investigation of these materials, partnered with a suitable supportive internal core, to create a multilayer composite material are needed to fully conclude on the suitability to produce a complete support structure. However, since many of the resulting properties of such materials are heavily dependent on the skin, these two candidates are likely to yield useful structural pieces with most core materials which are readily available.

The so-called mercerization process of bathing the fabrics in a concentrated solution of sodium hydroxide was found to have minimal impact on the resulting properties of the composite materials. Where a difference was seen, it did not result in an improvement of the final composite material in terms of strength or imaging performance. 

In all cases except for silk, the epoxy resins result in the highest flexural moduli. The soy-based UV curing resin, on the other hand, was found in this usage case to perform poorly and produce composite materials which are not well suited to applications in patient support for medical imaging and treatment. 

## 5. Conclusions

We have demonstrated through tensile testing, three-point bend assessment of flexural modulus and clinical imaging using MRI and X-ray that natural fibre composites represent an exciting alternative to the ubiquitous carbon fibre skin in exchange for a slight reduction in flexural modulus. 

The optimum materials were found to be unmercerized cotton or bamboo-based fibres infused with an epoxy or UV matrix respectively, providing minimal visibility on all modalities and a flexural modulus at 71% that of carbon fibre. 

Such materials will allow for the production of multimodal composite patient positioning devices thanks to their suitability for use in MRI and X-Ray applications with minimal impact on the resulting images. This will address a growing need in radiotherapy treatment planning for a truly multimodal patient positioning and support device. 

## 6. Patents

A patent has been filed (PCT/GB2020/050449) for the natural fibre composite materials with a manufacturer of patient positioning products, Medibord Ltd.

## Figures and Tables

**Table 1:** Details of matrix materials selected.

Material Name	Composition	Source	Planar Density
Silk	100% Habotai Silk	Midland Textiles, Birmingham, UK	35 gsm
Cotton	100% Egyptian Cotton	Sainsburys, London, UK	120 gsm
Lyocel	40% Lyocel 60% Cotton	IKEA, Milton Keynes, UK	120 gsm
Bamboo	100% Bamboo Fibre	Bamboo Panda Textiles, Cheshire, UK	160 gsm
Carbon Fibre	100% Carbon Fibre	EasyComposites, Staffordshire, UK	200 gsm

**Table 2:** Details of resins used in composite formation.

Resin Name	Type	Source	Curing Type
Epoxy	Infusion-IN2	Easy Composites, Staffordshire, UK	Two-Component chemical
Synthetic Resin	3D printer-FLGPWH01	Formlabs, Somerville, MA, USA	UV
Soy-based resin	3D printer–Eco UV Resin	Anycubic, Shenzhen, China	UV

## Supplementary Materials

The following are available online at https://www.mdpi.com/1996-1944/13/7/1684/s1, Figure S1: Comparison of treated and untreated samples for compressional testing, Figure S2: MRI data collected at 3T plotted as a function of flexural modulus for each of the material samples, Figure S3: Comparison between the tensile force of the fabric and the resulting flexural modulus of the composites.
