# Supplementary material for: Woven Natural Fibre Reinforced Composite Materials for Medical Imaging
Source: Materials (Basel). 2020 Apr 4;13(7):1684. doi: 10.3390/ma13071684 (PMC7178646; doi:10.3390/ma13071684)
Supplement: Supplementary file 1 [file materials-13-01684-s001.pdf]

Supplementary Information

Woven Natural Fibre Reinforced Composite Materials for Medical Imaging

Robert H. Morris [1,*], Nicasio R. Geraldi [1], Johanna L. Stafford [1], Abi Spicer [1], James Hall [1], Christopher Bradley [2] and Michael I. Newton [1]

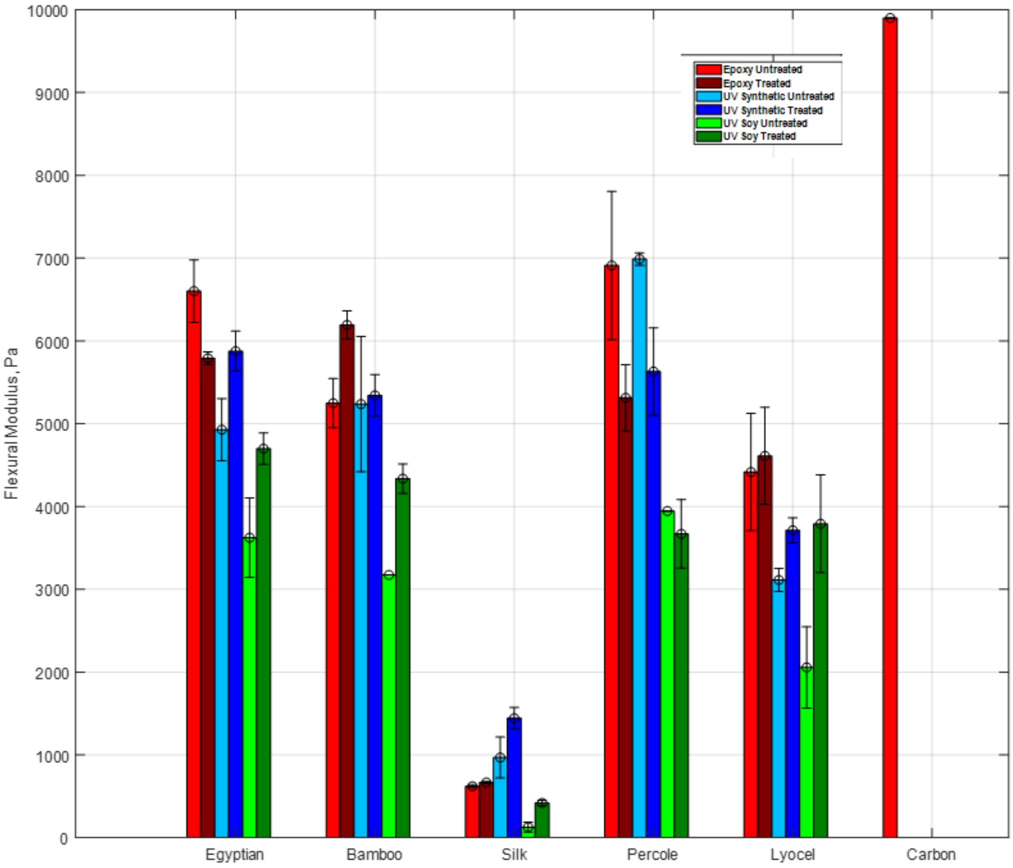

Figure S1. Comparison of treated and untreated samples for compressional testing. There is no significant difference between treated and untreated samples.

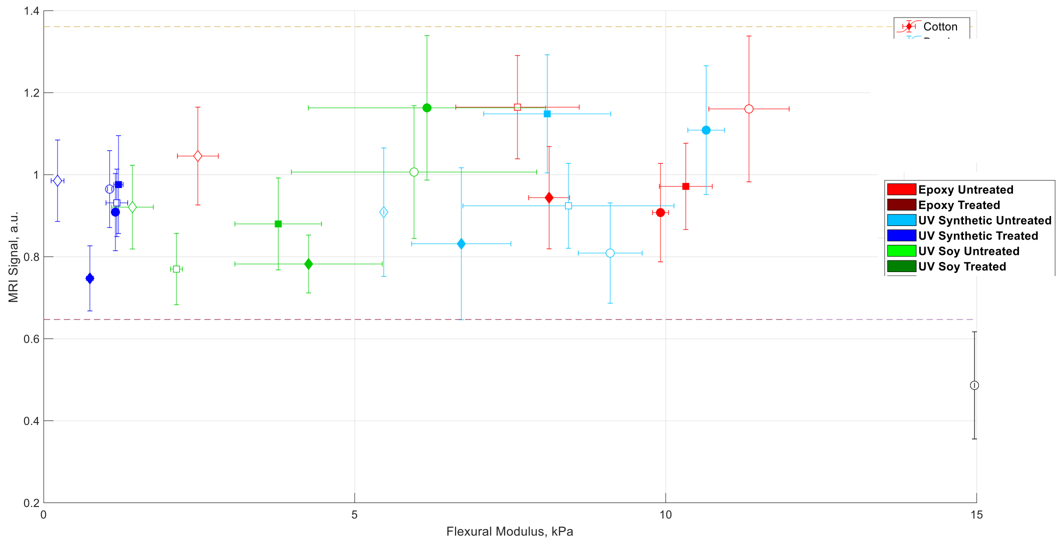

Figure S2. MRI data collected at 3T plotted as a function of flexural modulus for each of the material samples. Colours represent different fibres as detailed in the legend. The shapes represent different resins as follows: circles (O) Epoxy, Squares (□) Synthetic UV, Diamond (◇) Soy UV. Finally, untreated are open, and treated are filled. Whilst there is more scatter than present at 1.5T, the results all lie within the range of values found for the control solution only.

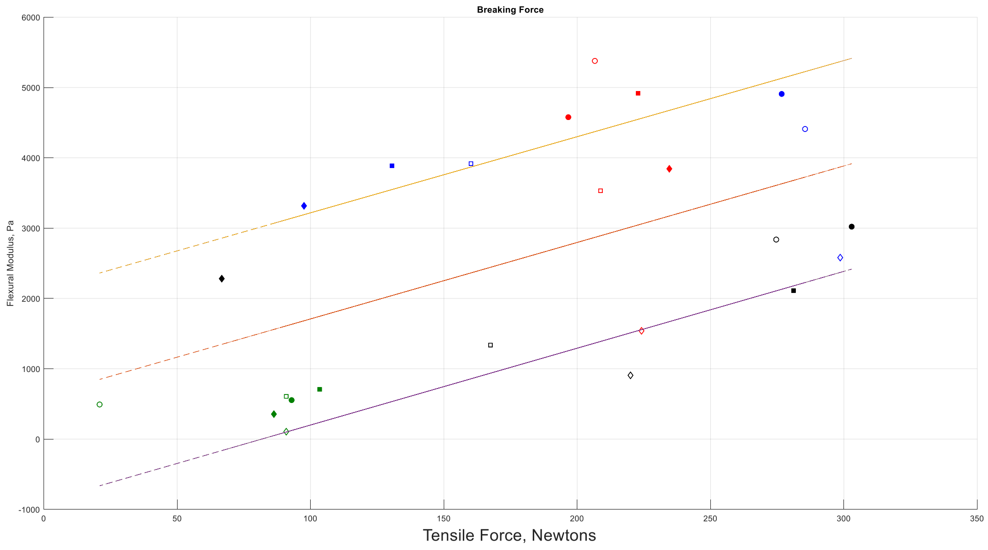

Figure S3. Comparison between the tensile force of the fabric and the resulting flexural modulus of the composites. The lines represent a linear fit and 50% confidence bars, demonstrating a trend, but with too much scatter to allow for use as a predictor of performance.
